# Acute effects of one-leg standing on arterial stiffness in older women: Role of the vision condition and standing dose

**DOI:** 10.3389/fphys.2022.1017486

**Published:** 2022-09-30

**Authors:** Zhixiong Zhou, Xiaoli Tao, Yuqin Zhang, Weili Zhu

**Affiliations:** Cardiovascular Health Laboratory, Capital University of Physical Education and Sports, Beijing, China

**Keywords:** one-leg standing, arterial stiffness, older women, vision, dose-response relationship

## Abstract

**Objective:** One-leg standing has been used exclusively for static balance testing and training purposes. We investigated the acute effects of one-leg standing with open or closed eyes on arterial stiffness in older women and explored the role of standing dose in arterial stiffness regulation.

**Methods:** Eighteen older women (60 ± 2 years) underwent non-intervention control (CON), one-leg standing with open eyes for 2 × 3 min (SO2), and one-leg standing with closed eyes for 1 × 3 min (SC1), 2 × 3 min (SC2), and 3 × 3 min trials (SC3) in a randomized self-controlled crossover fashion. Arterial stiffness in the cardio-ankle vascular index (CAVI) was measured at baseline (BL), immediately (0 min), and 10 and 20 min after standing. CAVI changes from BL in the same trial (⊿CAVI) were used for analysis.

**Results:** ⊿CAVI of the non-standing and standing side did not change with time in CON and SO2 trials. In SC1, SC2, and SC3 trials, ⊿CAVI of the standing side decreased significantly at 0 min compared to their corresponding BL (*p* < 0.01) and reverted gradually to the BL level afterward, with ⊿CAVI of the non-standing side undergoing no changes. At the time point of 0 min, only in the SC2 trial, ⊿CAVI of the standing side was significantly lower than that of CON (*p* < 0.01).

**Conclusion:** One-leg standing with closed eyes, but not with open eyes, resulted in transient arterial stiffness improvement in older women. The improvement was restricted to standing leg, and the moderate standing dose had maximal benefit on arterial stiffness.

## Introduction

Arterial stiffening is prominent in older people and could increase the risk of cardiovascular diseases. Strategies that combat the age- and sedentary-associated increase in arterial stiffness are of great importance. Compared to aerobic and resistance exercises, standing is more accessible in practice. However, current evidence does not support the efficacy of two-leg standing on arterial stiffness in humans (Caldwell et al., 2018; Greenlund et al., 2019).

As an ancient body-mind practice, yoga could reduce arterial stiffness in older people (Patil et al., 2015; Patil et al., 2020). Since yoga includes the posture of one-leg standing (OLS), it is reasonable to postulate that OLS, in contrast to bipedal standing, may exert the beneficial influence on arterial stiffness in older people.

OLS has long been exclusively adopted in static balance training and testing (Marcori et al., 2022). Visual information is an essential element for maintaining static balance (Bednarczuk et al., 2021), so OLS can be performed with either open or closed eyes, resulting in different muscle contraction levels (Patel et al., 2009; Muehlbauer et al., 2014). It would be interesting to examine to what extent the vision condition will influence the effects of OLS on arterial stiffness. Furthermore, considering that there was a dose–response relationship between the physical activity and health outcome (Garber et al., 2011), it is also pivotal to investigate whether the dose of OLS has an impact on arterial stiffness in older adults.

Therefore, this study aimed to examine the acute effects of OLS with eyes open or closed on arterial stiffness and investigated whether the dose of OLS influences the extent of arterial stiffness improvement in older adults.

## Methods

### Subjects and design

Eighteen healthy older women (age 60 ± 2 years) participated in the study (Table 1), with 11 of them having above-normal systolic blood pressure (i.e., ≥ 120 mm Hg). None reported any other diseases known to affect the cardiovascular system and the administration of oral contraceptives, antihyperlipidemic, antihypertensive, or antihyperglycemic medications. Informed consent was obtained from all subjects before the study. The Ethics Committee of the Capital University of Physical Education and Sports approved all procedures, and the study was carried out according to the Declaration of Helsinki.

**TABLE 1:** Subject characteristics.

Variable	Value (mean ± SEM)
Number of subjects	18
Number of subjects with above-normal systolic BP	11
Age (years)	60 ± 2
Age range (years)	48–75
Weight (kg)	62.9 ± 2.2
Height (cm)	158.6 ± 1.4
BMI (kg/m^2^)	25.1 ± 0.9
Systolic BP (mmHg)	124 ± 3
Diastolic BP (mmHg)	78 ± 1
CAVI of the standing side/non-standing side	8.2 ± 0.2/8.2 ± 0.2

Data are represented as means ± SEM.

BMI, body mass index; BP, blood pressure; CAVI, cardio-ankle vascular index.

**FIGURE 1:**
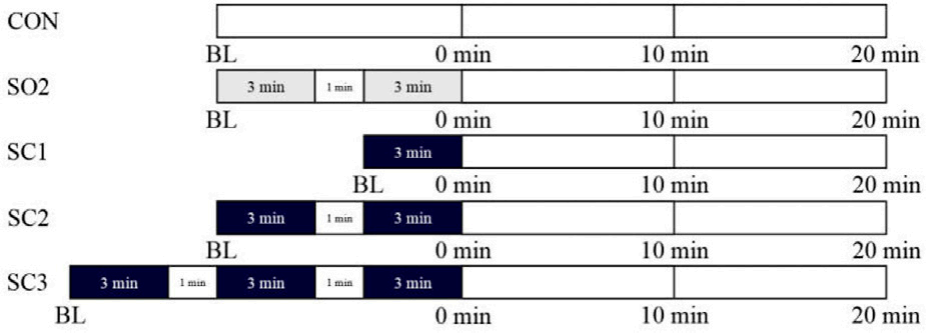
Study protocols for one-leg standing, rest interval, visual condition, and measurements in five trials: non-standing CONtrol (CON), one-leg standing with open eyes for 2 × 3 min (SO2), and one-leg standing with closed eyes for 1 × 3 min (SC1), 2 × 3 min (SC2), and 3 × 3 min trials (SC3). Measurements were performed at baseline (BL), immediately (0 min), 10, and 20 min after one-leg standing. The gray area indicates one-leg standing with open eyes. The black area indicates one-leg standing with closed eyes.

All participants underwent five trials including non-standing control (CON), one-leg standing with eyes open for 2 × 3 min (SO2), and one-leg standing with eyes closed for 1 × 3 min (SC1), 2 × 3 min (SC2), and 3 × 3 min trials (SC3) in a randomized self-controlled crossover fashion (Figure 1). In the CON trial, participants remained seated in chairs quietly for 7 min, while in all other trials, they performed standing with eyes open or closed as illustrated in Figure 1. Every two consecutive trials for each participant were separated by a 7-day washout period to eliminate the residual effects of the former trial.

All participants abstained from vigorous activity and alcohol or caffeine intake over the day before each trial. Participants entered their own trials once a week for five consecutive weeks on the same timing of the day, fasting at least for 3 h, and rested quietly for at least 15 min before baseline measurement. All the trials were conducted with room temperature ranging from 22 to 25°C in the Cardiovascular Health Laboratory of the Capital University of Physical Education and Sports.

### One-leg standing

The participant was asked to stand on their left leg, with eyes either open or closed for single or multiple 3-min bouts whilst flexing the contralateral knee. The left leg was chosen for all the participants in all the standing trials, for the dominant and the non-dominant leg can be used interchangeably during static one-leg balance testing (Muehlbauer et al., 2014). All the standing was performed on the firm ground in the laboratory. The bout of standing length of was fixed at 3 min because it was the upper limit for most participants in our preliminary test. The interval between the adjacent 3-min bouts was 1 min, somewhat arbitrary, for the purpose of participant’s rest and recovery. It was hard for participants to achieve 3-min bout continuously, and multiple attempts were allowed to accumulate toward the 3 min. Most subjects experienced body sway, especially during the late period of the 3-min bout, and the examiner stood close to the participant to give the slightest touch to prevent falls and fall injuries.

### Measurements

Using a VaSera 1500 vascular screening system (Fukuda Denshi, Beijing, China), arterial stiffness was evaluated in the cardio-ankle vascular index (CAVI), an index of systemic arterial stiffness that reflects the condition of the aorta, femoral artery, and tibial artery. With the subjects in the supine position, electrocardiogram electrodes were placed on wrists, a microphone for monitoring heart sounds (phonocardiogram) was placed on the sternum, and four cuffs were wrapped around the upper arms and ankles. When the electrocardiogram was stable and the first and second heart sounds were detected in the phonocardiogram, the START button was pressed, and the values of right and left CAVI were obtained using the system completely independent of human operation.

Measurements of blood pressure and heart rate were made simultaneously by the system.

### Statistical analysis

Statistical processing was performed using GraphPad Prism Software 8.0. All data are expressed as means ± SEM. CAVI changes from BL in the same trial (⊿CAVI) were used for analysis. The responses of ⊿CAVI, HR, and BP to exercise were analyzed by two-way (treatment × time) ANOVA of repeated measures. A *post hoc* test was performed to probe the time point at which the difference between trials existed and to identify the significant changes with time within the trial. The statistical significance level was set at *p* < 0.05.

## Results

Table 1 shows the subjects’ baseline characteristics including age, height, weight, body mass index, blood pressure, and arterial stiffness. Eleven of the subjects were with above-normal systolic blood pressure, and the arterial stiffness of standing and non-standing was of no difference.

**FIGURE 2:**
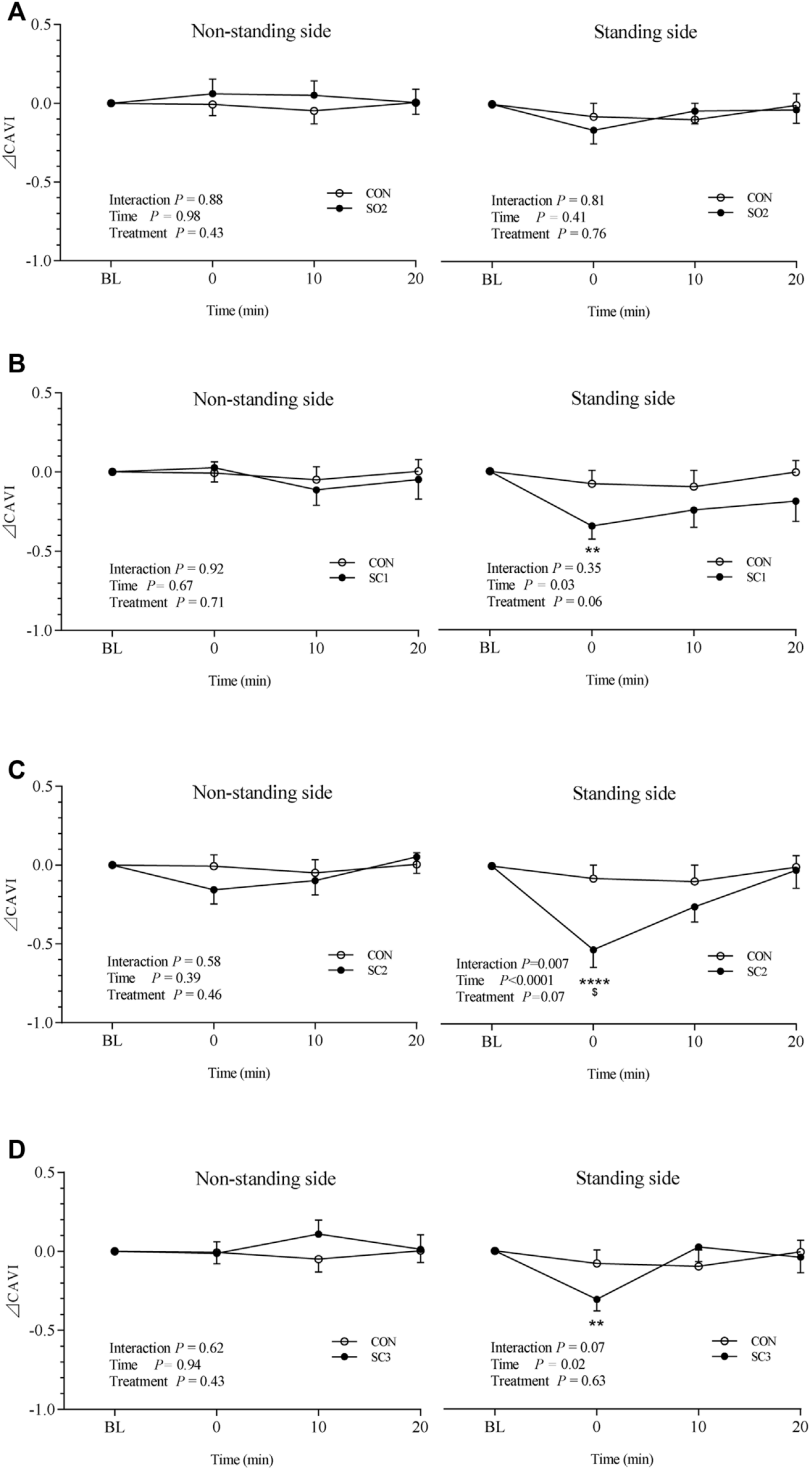
Mean (± SEM) time-dependent ⊿CAVI changes of both the non-standing side and standing side undergoing CON and SO2 trials **(A)**, CON and SC1 trials **(B)**, CON and SC2 trials **(C)**, and CON and SC3 trials **(D)**. Statistical analysis was performed by using a two-factor (treatment and time) ANOVA with repeated measures with Bonferroni *post hoc* tests. Data are represented as means ± SEM, *n* = 18. ^**^ *p* < 0.01 vs. BL within the same trial, ^****^ *p* < 0.0001 vs. BL and 20 min within the same trial, and ^$^ *p* < 0.01 vs. CON trial at the same time point. CON = non-standing control; SO2 = one-leg standing with open eyes for 2 × 3 min; SC1 = one-leg standing with closed eyes for 1 × 3 min; SC2 = one-leg standing with closed eyes for 2 × 3 min; SC3 = one-leg standing with closed eyes for 3 × 3 min.

As can be seen in Figure 2, ⊿CAVI of the non-standing side remained stable with time in the CON trial and all one-leg standing trials. As to the standing side, ⊿CAVI remained constant with time in both CON and SO2 trials (Figure 2A). In the SC1 trial, however, ⊿CAVI of the standing side decreased significantly from 0.0 ± 0.0 at BL to -0.3 ± 0.1 at 0 min (^**^ *p* < 0.01 vs. BL, Figure 2B) and to -0.5 ± 0.1 at 0 min (^****^ *p* < 0.0001 vs. BL, 10, and 20 min, Figure 2C) in the SC2 trial and to -0.3 ± 0.1 at 0 min (^**^ *p* < 0.01 vs. BL, 10, and 20 min, Figure 2D) in the SC3 trial. At the time point of 0 min, ⊿CAVI of the SC2 trial was significantly lower than that of the CON trial (^$^ *p* < 0.01 vs. CON trial at the same time point, Figure 2C).

Specifically, ⊿CAVI of the standing side remained unchanged with time in both CON and SO2 trials (Figure 2A). In the SC1 trial, ⊿CAVI was 0.0 ± 0.0, -0.3 ± 0.1, -0.2 ± 0.1, and -0.2 ± 0.1 at BL, 0, 10, and 20 min, respectively (Figure 2B). In the SC2 trial, ⊿CAVI was 0.0 ± 0.0, -0.5 ± 0.1, -0.3 ± 0.1, and 0.0 ± 0.1 at BL, 0, 10, and 20 min, respectively (Figure 2C). In the SC3 trial, ⊿CAVI was 0.0 ± 0.0, -0.3 ± 0.1, 0.0 ± 0.1, and 0.0 ± 0.1 at BL, 0, 10, and 20 min, respectively (Figure 2D).

As seen in Table 2, the heart rate and brachial diastolic blood pressure did not change significantly after standing. However, brachial systolic blood pressure increased significantly at 0 min and returned to the baseline level at 20 min in the SC3 trial, remaining unaltered with time in all other standing trials. In the SC2 trial, brachial systolic blood pressure decreased significantly at 20 min compared to 0 min.

**TABLE 2:** Effects of one-leg standing on heart rate and brachial artery blood pressure (*n* = 18).

	BL	0 min	10 min	20 min
HR (beats/min)				
CON	60 ± 2	62 ± 2	60 ± 2	60 ± 2
SO2	62 ± 3	65 ± 3	62 ± 3	61 ± 3
SC1	62 ± 2	64 ± 2	61 ± 2	64 ± 4
SC2	64 ± 3	64 ± 2	61 ± 2	62 ± 2
SC3	62 ± 2	66 ± 2	63 ± 2	62 ± 2
SBP (mmHg)				
CON	122 ± 2	123 ± 2	125 ± 3	127 ± 3
SO2	124 ± 4	130 ± 3	127 ± 3	127 ± 3
SC1	124 ± 2	128 ± 3	125 ± 3	124 ± 3
SC2	127 ± 3	131 ± 4	126 ± 3	125 ± 4^&^
SC3	127 ± 3	133 ± 3^*^	128 ± 3	127 ± 3
DBP (mmHg)				
CON	77 ± 1	78 ± 1	80 ± 1	80 ± 2
SO2	77 ± 2	80 ± 2	79 ± 2	80 ± 2
SC1	76 ± 1	79 ± 1	79 ± 1	79 ± 2
SC2	78 ± 1	80 ± 2	80 ± 1	78 ± 1
SC3	78 ± 2	82 ± 1	80 ± 1	79 ± 1

Values are represented as mean ± SEM.

CON = non-standing control; SO2 = one-leg standing with open eyes for 2 × 3 min; SC1 = one-leg standing with closed eyes for 1 × 3 min; SC2 = one-leg standing with closed eyes for 2 × 3 min; SC3 = one-leg standing with closed eyes for 3 × 3 min; HR = heart rate; SBP = systolic blood pressure; DBP = diastolic blood pressure.

^*^ *p* < 0.05 vs. BL and 20 min within the SC3 trial.

^&^ *p* < 0.05 vs. 0 min within the SC2 trial.

As indicated in Table 3, both ankle blood pressure and ABI remained unaltered in standing and non-standing sides.

**TABLE 3:** Effects of one-leg standing on ankle blood pressure and ABI (*n* = 18).

	BL	0 min	10 min	20 min
SBP_St (mmHg)				
CON	137 ± 2	142 ± 3	141 ± 3	144 ± 3
SO2	141 ± 3	142 ± 3	146 ± 3	145 ± 3
SC1	140 ± 3	141 ± 3	140 ± 3	141 ± 3
SC2	143 ± 3	143 ± 3	142 ± 3	142 ± 3
SC3	144 ± 3	147 ± 3	144 ± 3	142 ± 2
DBP_St (mmHg)				
CON	74 ± 1	74 ± 1	75 ± 1	77 ± 1
SO2	74 ± 2	73 ± 1	76 ± 2	76 ± 2
SC1	73 ± 1	72 ± 1	74 ± 1	75 ± 1
SC2	74 ± 1	74 ± 1	76 ± 1	75 ± 1
SC3	75 ± 1	74 ± 1	76 ± 1	76 ± 1
SBP_NSt (mmHg)				
CON	137 ± 3	138 ± 3	142 ± 3	143 ± 3
SO2	140 ± 4	144 ± 3	144 ± 3	143 ± 3
SC1	139 ± 3	141 ± 3	138 ± 3	140 ± 3
SC2	142 ± 3	145 ± 3	142 ± 3	141 ± 3
SC3	142 ± 4	147 ± 4	144 ± 3	143 ± 3
DBP_NSt (mmHg)				
CON	72 ± 2	72 ± 1	75 ± 1	75 ± 1
SO2	72 ± 2	74 ± 2	75 ± 2	75 ± 1
SC1	70 ± 1	73 ± 2	73 ± 1	75 ± 1
SC2	74 ± 1	75 ± 2	75 ± 2	75 ± 1
SC3	72 ± 2	76 ± 1	76 ± 1	75 ± 1
ABI_St				
CON	1.11 ± 0.02	1.14 ± 0.02	1.12 ± 0.01	1.13 ± 0.02
SO2	1.14 ± 0.02	1.10 ± 0.02	1.14 ± 0.02	1.13 ± 0.01
SC1	1.13 ± 0.02	1.11 ± 0.02	1.13 ± 0.02	1.14 ± 0.02
SC2	1.14 ± 0.02	1.09 ± 0.02	1.14 ± 0.02	1.14 ± 0.02
SC3	1.14 ± 0.01	1.11 ± 0.01	1.12 ± 0.02	1.13 ± 0.02
ABI_NSt				
CON	1.10 ± 0.02	1.10 ± 0.02	1.13 ± 0.02	1.12 ± 0.01
SO2	1.13 ± 0.02	1.11 ± 0.02	1.12 ± 0.02	1.10 ± 0.01
SC1	1.12 ± 0.02	1.10 ± 0.01	1.11 ± 0.01	1.13 ± 0.01
SC2	1.13 ± 0.01	1.11 ± 0.02	1.14 ± 0.01	1.13 ± 0.02
SC3	1.11 ± 0.02	1.11 ± 0.02	1.12 ± 0.01	1.13 ± 0.01

Values are represented as mean ± SEM.

CON, non-standing control; SO2, one-leg standing with open eyes for 2 × 3 min; SC1, one-leg standing with closed eyes for 1 × 3 min; SC2, one-leg standing with closed eyes for 2 × 3 min; SC3, one-leg standing with closed eyes for 3 × 3 min; SBP, systolic blood pressure; DBP, diastolic blood pressure; St, standing side; NSt, non-standing side; ABI, ankle brachial index.

## Discussion

Our findings are that OLS with closed eyes, in contrast to open eyes, acutely improved arterial stiffness of the standing side in older women. However, this transient effect was attenuated when the dose of OLS was either increased or decreased.

### Effects of bipedal vs. monopedal standing on arterial stiffness

Evidence demonstrates that sedentary time is positively associated with arterial stiffness (Horta et al., 2015), and sedentary behavior interruption helps reduce arterial stiffness. To interrupt sedentary behavior, standing is more feasible than aerobic and resistance exercises, especially in the office environment. However, the cross-sectional study showed no significant difference in arterial stiffness between the sitting group and two-leg standing group (Greenlund et al., 2019); and to the worse, acute prolonged two-leg standing for 2 h even increases arterial stiffness (Caldwell et al., 2018). Another acute study showed that two-leg standing alternating sitting every 30 min suppressed the arterial stiffness increase induced by sitting throughout the day (Barone Gibbs et al., 2017). Till now, no studies ever reported that bipedal standing could improve the participants’ arterial stiffness from their baseline level.

Compared to bipedal standing, OLS is more challenging due to the narrower support base (Suponitsky et al., 2008) and the limited haptic sensory information from one leg rather than two legs. Sensory input, including haptic sensory information, can affect muscle activation and hence determine the biomechanical properties of muscles (Dickerson 2020), and people could decrease muscle contraction using the light fingertip contact (Iwamoto et al., 2017). So, it is not surprising that muscular activity during OLS is more intensive (Suponitsky et al., 2008). However, the present study found that two bouts of 3-min OLS with open eyes could not improve arterial stiffness in older women. This indicates that muscular activity during OLS is not intensive enough to constitute a sufficient stimulus to modulate the arterial stiffness in older people.

### Effects of vision on arterial stiffness and blood pressure response induced by one-leg standing

Vision is one of the elements of the system responsible for maintaining static balance and provides important sensory information required by balancing. Any visual impairments or conditions hampering the reception of visual stimuli may affect the static balance. Mechanical instability needs to be counterbalanced by the precise activation of multiple muscles (Danna-Dos-Santos et al., 2015), and thus vision plays a key role in the generation of common inputs to the muscles, and its condition could be used to vary the task difficulty level of standing. In this study, we hypothesized that closed-eye-induced intensive muscle activity would alter the arterial stiffness response to OLS.

Our results showed that after OLS with closed eyes, no matter what the bout number is, the arterial stiffness of the standing side in the older women improved significantly, though transiently. In our study, the legs experienced the same hydrostatic pressure due to gravity in both closed- and open-eye trials, and thus, one-leg standing-induced shear stress did not significantly differ between both trials. So, it seems unlikely that hydrostatic pressure led to the impact of one-leg standing with closed eyes on arterial stiffness. Considering that intensity is an important determinant of the physiological responses to exercise training (Garber et al., 2011), we proposed that, compared to two-leg standing and OLS with open eyes, the relatively high intensity of muscle isometric contraction of OLS with closed eyes might account for the significant arterial stiffness changes. Previous evidence shows that muscles were more involved when OLS was performed with eyes closed than with eyes open (Patel et al., 2009; Muehlbauer et al., 2014). During OLS with closed eyes, it is very likely that static leg muscle contraction elicited threshold intramuscular pressure and resultant blood flow occlusion (Oranchuk et al., 2020), constituting contrast to dynamic exercise. The attenuated removal of metabolites during isometric contraction (Greaney et al., 2015) may play a critical role in the regulation of leg arterial stiffness.

In this study, the mild but still significant improvement of arterial stiffness in terms of CAVI induced by closed-eye OLS is comparable to that observed in acute aerobic exercise (Masaki et al., 2019) and long-term Swiss ball exercise (Ikebe et al., 2022), demonstrating the efficacy of OLS protocol in practice. This relatively short acute response is also of importance, for some of the major health-related changes produced by physical activity may be due to more “acute” biological responses during and for some time following each bout of activity than a “training” response (Haskell 1994). In addition, there may be an interaction between these two types of responses, for some acute response may be augmented by repeated bouts of exercise (Haskell 1994). Whether the effects of repeated bouts of OLS with closed eyes on arterial stiffness could yield “training” effects deserve investigation in the future study.

However, Sugawara et al. (2010) reported that arterial stiffness of the exercised leg did not change after exercise in older subjects. The nature of exercise adopted might explain the conflicting findings between the results of our study and Sugawara et al. It is likely that the arterial stiffness response in older subjects is more sensitive to OLS than to low-intensity cycling. During OLS, the leg muscles work primarily in an isometric manner, during which the muscles are more likely to be subjected to metabolite accumulation (Greaney et al., 2015). Another factor responsible for the absence of arterial stiffness response to cycling is the small number of subjects involved (Sugawara et al., 2010), which could not eliminate the possibility that the heterogeneity of subjects resulted in a profile of unchanged arterial stiffness to cycling.

In this study, brachial systolic blood pressure tends to increase immediately after standing overall and reached significance after three bouts of standing with closed eyes. This supports the notion that heart rate and blood pressure increase in order to acutely meet the metabolic demands of the working skeletal muscle during exercise (Greaney et al., 2015) and indicated that standing volume play an important role in the regulation of blood pressure. Though arterial stiffness decreased immediately after standing with closed eyes, ankle blood pressure remained unaltered. This again proved that CAVI is an arterial stiffness parameter that is not influenced by blood pressure (Saiki et al., 2016).

### Standing dose and arterial stiffness improvement

Guidelines recommended that older people participate in aerobic and muscle-strengthening activities above minimum recommended amounts to gain additional health benefits and higher levels of physical fitness (Nelson et al., 2007). Similarly, greater balance improvement effects can be seen in programs that include exercises that challenge balance and use a higher dose of exercise (Sherrington et al., 2008). However, these guidelines assumed that people are all sedentary and reluctant to pursue physical activity, neglecting and failing to set an upper limit of certain kinds of activities for active people for the health purpose. Thus, it is of importance to know the optimal OLS dose for the arterial stiffness improvement in older people. Based on the efficacy of OLS with closed eyes, we further explored the effects of standing dose, though within a very narrow dose range, on arterial stiffness regulation.

The results showed that, though all OLS protocols with closed eyes have significant impact on arterial stiffness in older women, the optimal protocol is two bouts of 5-min OLS, and the attenuation of the impact was observed when the OLS bout number was either increased to three or decreased to one. Specifically, an increase in OLS dose within a very narrow range (from one to two bouts) produced augmented response. This reminds us that caution should be taken on the dose of OLS in practice to ensure that OLS can have maximal health benefit. Furthermore, whether a fourth or more bouts will deteriorate, and even abolish, the arterial stiffness improvement induced by OLS warrants further investigation.

The phenomenon that a third bout of OLS attenuates the impact of previous two bouts is very similar to that of ischemic conditioning. In dose–response studies, researchers found that an increased number of ischemic cycles could attenuate, or even abolish, the beneficial effects that a certain number of cycles produced (Iliodromitis et al., 1997; Zahir et al., 1998). This was summarized and termed as “hyperconditioning” by Whittaker and Przyklenk, (2014). Based on these facts, we proposed that OLS and ischemic conditioning share characteristics of hormesis, i.e., a biphasic dose–response curve (Calabrese 2013). Specifically, the benefits are found at low doses, whereas loss of these benefits and even adverse effects occur at high doses.

### Localized effects of one-leg standing on arterial stiffness

Previous studies demonstrated that only arterial stiffness in the exercised leg decreased after acute aerobic exercise (Sugawara et al., 2003), resistance exercise (Heffernan et al., 2006), and static passive stretching (Yamato et al., 2017), while central and non-exercised leg arterial stiffness remained unaltered. Our study supported this local effect of exercise from the perspective of static standing, showing that CAVI of the standing side decreased after OLS, whereas CAVI of the non-standing side remained unchanged. Since CAVI is an index of systemic arterial stiffness that reflects the condition of the aorta, femoral artery, and tibial artery, and the aorta is the shared part that constitutes the measurement results of both left and right CAVI, the difference between exercised and non-exercised CAVI could only be ascribed to the different arterial stiffness conditions between the arteries of the standing leg and non-standing leg. As typical localized static exercise involving mainly muscles on the standing leg, OLS seems to elicit regional rather than systemic factors that account for the arterial stiffness response.

## Limitations

First, we enrolled older women with both normal and above-normal blood pressure; however, we used self-control design in this study, and the results indicated that the results might apply to people with both normal and above-normal blood pressure. Second, we, somewhat arbitrarily, fixed the interval between standing bouts at 1 min in this study. Since the interval between cycling bouts may influence arterial stiffness improvement (Zheng et al., 2015), whether this is also the case with standing deserves investigation. Finally, this is an acute intervention study, and the arterial stiffness changes induced by OLS remained relatively short. Since the health benefit of an “acute” exercise response does not necessarily result in that of a “training” response (Haskell 1994), future studies are required to examine the long-term impact of regular OLS training with closed eyes and offer a mechanistic insight.

## Conclusion

Though OLS with open eyes was not a sufficient stimulus to elicit an arterial stiffness decrease in older women, OLS with closed eyes could transiently improve arterial stiffness in the standing side but not the non-standing side. Moderate standing dose was necessary in order to benefit arterial stiffness the most. Our study provided an easily accessible protocol for older women to condition arterial stiffness at any time when they wanted to exercise, and this will contribute to the improvement of cardiovascular health and the prevention of cardiovascular diseases of older women.

## Data Availability

The raw data supporting the conclusion of this article will be made available by the authors, without undue reservation.
